# Prevalence of QTc Prolongation in Patients with Parkinson’s Disease. Assessment of the Effects of Drugs, Clinical Risk Factors and Used Correction Formula

**DOI:** 10.3390/jcm10071396

**Published:** 2021-03-31

**Authors:** Jakub J. Malkiewicz, Maciej Malkiewicz, Joanna Siuda

**Affiliations:** 1 Department of Neurology, Faculty of Medical Sciences in Katowice, Medical University of Silesia in Katowice, University Clinical Center Prof. K. Gibiński, 14 Medyków Street, 40-752 Katowice, Poland; jakub.jerzy.malkiewicz@gmail.com; 2 Department of Gastroenterology with Subdivision of Internal Medicine, John Paul II Memorial Beskid Center of Oncology—Municipal Hospital in Bielsko Biala, 21 Stanisława Wyspiańskiego Street, 43-300 Bielsko-Biała, Poland; macmal3@o2.pl

**Keywords:** Parkinson’s disease, ECG, QTc prolongation, risk factors, drugs, age, sex, dysautonomia, autonomic nervous system

## Abstract

Background: Parkinson’s disease (PD) is a possible risk factor for corrected QT interval (QTc) prolongation. PD patients frequently take QTc-prolonging drugs. The aim of the study was to assess the prevalence of QTc prolongation in PD and the influence of drugs and other potential risk factors on the QTc length in PD. Methods: 101 patients with PD and a good quality ECG were included in the study. The prolonged QTc was defined as ≥450 ms for men and ≥460 ms for women. Bazett’s (QTcB) and Framingham (QTcF) formulas were utilized to calculate QTc. Data about sex, age, PD duration, disease’s severity, comorbidities and QTc-prolonging drugs were collected. Multiple linear regressions with backward elimination were used to assess factors influencing the QTc. Results: A long QTc was presented in 13 patients (12.9%) for QTcB and 4 patients (4%) for QTcF. Longer QTc in PD patients was associated with older age, male sex and QTc-prolonging drugs regardless of the used formula. The QTcB was also significantly affected by the heart rate (HR). Conclusion: QTc prolongation is common in PD. Age, drugs and male gender are potential risk factors for QTc prolongation in PD.

## 1. Introduction

PD is a neurodegenerative disorder characterized by motor and nonmotor symptoms like hyposmia, depression, hallucinations, cognitive impairment, sleep disorders, as well as sympathetic and parasympathetic autonomic nervous system dysfunction [1]. Cardiac sympathetic denervation which could be shown in [123I] metaiodobenzylguanidine (MIBG) scintigraphy or 6-[18F] fluorodopamine (6-FD) positron emission tomography is presented in PD [2]. PD patients also present parasympathetic abnormalities in heart regulation [1,2]. Clinical symptoms of cardiovascular autonomic dysfunction include orthostatic hypotension, postprandial hypotension, nocturnal supine hypertension, impaired baroreflexes and diminished heart rate variability [1,2,3]. There are also some data suggesting that PD might predispose patients to heart failure and sudden cardiac death [3,4]

QTc prolongation is a risk factor for torsade de pointes (TdP) and ventricular fibrillation, which can result in syncope and sudden cardiac death. A few studies indicate that people suffering from PD have a longer QTc or more often have QTc prolongation than healthy controls, which is probably associated with autonomic nervous system dysfunction [4,5,6,7]. Acquired QTc prolongation is frequently associated with medication including many drugs commonly used in Parkinson’s disease like antidepressants, antiemetics, atypical antipsychotics and antidementia agents. Medication seems to play an important role in QTc prolongation in PD [8,9,10]. Cunnington et al. have found high prevalence of QTc prolongation in PD, especially in patients taking QTc-prolonging drugs [8]. On the other hand, in a cross-sectional study by J. Piqueras-Flores et al., there was no significant difference between patients taking QTc-prolonging drugs and patients who did not take them [4]. Patients with more advanced PD had longer QTc intervals in some studies [5,6,8,11]. Almost all previous studies used only the most popular, but imperfect, QTcB for heart rate correction. It is known that QTcB overcorrects the QTc interval duration at high HR and undercorrects it at low HR [12,13]. The consequence of using this formula is an overestimation of the number of patients with potentially dangerous QTc prolongation even if HR was <90 beats per minute, which might result in unnecessary drugs withdrawal [12,13]. Other formulas like Fridericia, QTcF or Hodges should be preferred [12,13]

The aims of this study were to assess the prevalence of potentially dangerous QTc prolongation, estimation of bias caused by using QTcB and evaluation of factors associated with QTc prolongation in patients with Parkinson’s disease.

## 2. Materials and Methods

### 2.1. Population and QTc Assessment

Patients with the diagnosis of PD according to current MDS criteria and an available good quality standard ECG were prospectively included in the study [14]. All patients were recruited in a single site, a tertiary reference neurology center, and were examined by the same movement disorders specialist (JS). Information about age, sex, disease duration, QTc-prolonging drugs, kalemia, comorbidities, disease severity in Hoehn-Yahr staging (HYs), as well as the third part of UPDRS in OFF and ON states, were collected. Patients with bundle branch block, hypokalemia and abnormal thyrotropin level were excluded. The QT-prolonging drugs list was obtained from www.crediblemeds.org [15]. The list for three torsade de pointes risk categories (known, possible and conditional) was utilized in the study to define QTc-prolonging drugs.

The duration of the QT interval was independently measured by two observers on all leads in 3 heart cycles when the rhythm was regular and 5 cycles if the rhythm was irregular. Both observers (JM and MM) were blind for patients’ clinical data at the moment of the assessment. Measurements were averaged for every lead, and the lead with the longest mean QT was used for calculation of QTc. Next, the results for both observers were averaged and inter-observer variability was calculated. Two formulas were used to compute QTc:

- QTcB = QT/RR^1/2^
- QTcF = QT + 0.154(1−RR).

The prolonged QTc was defined according to AHA/ACCF/HRS Recommendations and Polish Cardiac Society (PCS) Recommendations as ≥450 ms for male and ≥460 ms for female patients [12,16].

### 2.2. Statistical Analysis

STATISTICA 13 PL software (Tibico Software Inc.) was used for data analysis. All used tests were two-tailed. Nominal data were presented as a number and percentages. Continuous variables were presented as a mean ± standard deviation in the case of normal distribution or median (lower quartile-upper quartile) when distribution was not normal. Shapiro–Wilk test and Q-Q plots were used to test normality. Inter-observer reliability and agreement between both used formulas in recognition of QTc prolongation were assessed by Coehn’s kappa coefficient. Cohen’s kappa was interpreted according to Landis et al. (<0.00: poor, 0.00–0.20: slight, 0.21–0.40: fair, 0.41–0.60: moderate, 0.61–0.80: substantial, 0.81–1: almost perfect) [17]. Bland–Altman plot with calculation limits of agreement and mean difference was also used to show difference between QTcB and QTcF. Firstly, Mann–Whitney U test, Student’s t test, Spearman’s rank (R_S_) and Pearson’s correlation (R_P_) were utilized to assess the effects of analyzed factors. Next, factors with *p* < 0.50 were included in the multivariable linear regression model with backward elimination, which was used to assess factors influencing the QTc length calculated with both formulas. Continuous predictors were centered on mean and effect coding (−1, 1) and were used for binary predictors. The coefficient of partial determination (r_p_^2^) was utilized to assess the effect size of significant predictors in the regression model. The level of statistical significance was set at *p* < 0.05.

### 2.3. Ethical Considerations

The study was registered with the Bioethical Committee of the Medical University of Silesia in Katowice, Poland (Letter PCN/0022/KB/42/20).

## 3. Results

112 PD patients, who had been referred for the assessment of motor and nonmotor symptoms, and had available good quality ECG, were admitted to the Neurology Department. After negative verification of PD diagnosis and exclusion of the patients with bundle branch block, thyroid dysfunction and hypokalemia, 101 patients were finally included, and their characteristics are presented in Table 1.

56 (55%) patients took one or more QTc-prolonging drugs, but only 10 (9.9%) and 12 (11.9%) patients took drugs from definite and possible categories of TdP risk, respectively. Drugs classified as a conditional risk category of TdP were taken by 47 (46.5%) patients. 22 (22%) patients took two or more QT-prolonging drugs. The number of drugs from particular categories used in the studied group of patients and mean doses is presented in Table 2.

**Figure 1:**
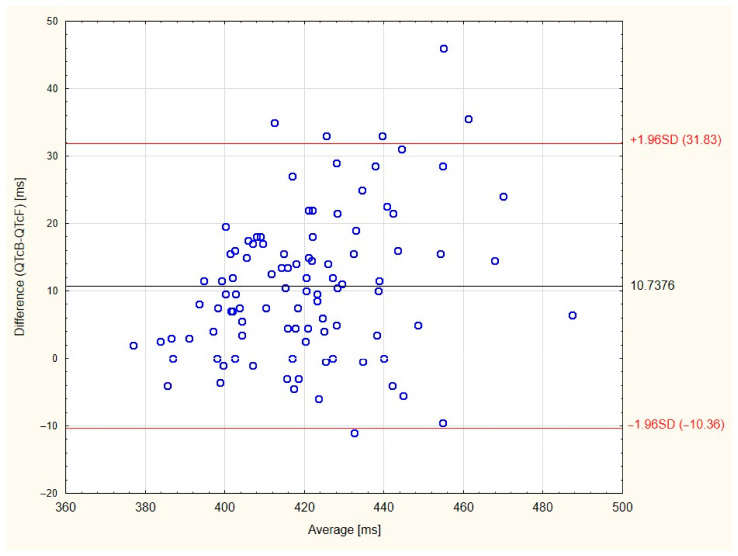
Bland–Altman diagram for QTcB and QTcF. Note: Black line-mean difference between QTcB and QTcF, red lines-95% limits of inter-rater agreement.

QTc prolongation was presented in 13 (12.9%) patients for QTcB and four (4.0%) for QTcF. None of them had QTc longer than 500 ms, which is considered to be connected with especially high risk of torsade de pointes. Investigators assessing ECGs inter-rater agreement rate was 93% for QTcB (kappa 0.68 SE = 0.11) and 98% for QTcF (kappa 0.79 SE = 0.15)—which means substantial agreement. For QTc formulas, inter-rater agreement rate was 91% (kappa 0.44 SE = 0.15)—meaning moderate agreement. Mean difference between QT calculated with both formulas was 10.74 ± 10.76 ms, 95% limits of agreement −10.36 to 31.83 ms (Figure 1). All patients with QTcF prolongation and 10 patients with QTcB prolongation were taking at least one QTc-prolonging drug. Characteristics of patients with QT prolongation according to both formulas is presented in Table 3.

The influence of factors potentially associated with QTc prolongation was assessed by bivariate analysis (Table 4). The QTc interval duration was significantly correlated with age regardless of the used formula (QTcB R_P_ = 0.29, *p* = 0.003; QTcF R_P_ = 0.32, *p* = 0.001). HR was significantly correlated with QTcB (R_P_ = 0.42, *p* < 0.001), but not with QTcF (R_P_ = 0.06, *p* = 0.576). A significant difference was found between patients with heart diseases (ischemic heart disease and/or heart failure) and without them (QTcB 439.1 ± 24.2 vs. 423.0 ± 21.2 *p* = 0.008; QTcF 426.3 ± 21.8 vs. 412.6 ± 18.0, *p* = 0.008). Males had significantly longer QTc according to QTcF (417.7 ± 20.6 vs. 409.5 ± 15.1, *p* = 0.026)—however, not in the case of QTcB (427.0 ± 24.5 vs. 422.7 ± 17.9, *p* = 0.312). Gender, age, UPDRS ON, HYs, hypertension, heart diseases, the use of QTc-prolonging drugs and HR only for QTcB were included in the regression analysis. UPDRS OFF, PD duration, presence of diabetes for each formula and HR for QTcF did not reach *p*-value below < 0.50 and were not considered in the multiple linear regression analysis.

Multiple linear regression with backward elimination revealed that age, male sex and QT-prolonging drugs are predictive of the QT length in PD patients, regardless of the used formula. QTcB was also significantly affected by HR (Table 5). Both final models were significant: QTcB F(4,96) = 12.78; *p* < 0.001, QTcF F(3,97) = 8,353; *p* < 0.001 and explained 31.9% (R^2^ = 0.347, adjusted R^2^ = 0.319) and 18.1% (R^2^ = 0.205, adjusted R^2^ = 0.181) of variance, respectively. Predictors which explained the greatest part of variance were HR for QTcB (r_p_^2^ = 0.240) and age for QTcF (r_p_^2^ = 0.13). In both models, effect size of age was larger than male gender, and the use of QT-prolonging drugs explained the least part of variance (Table 5).

## 4. Discussion

In this study we assessed the prevalence of QTc prolongation and factors influencing the QTc interval duration in patients suffering from Parkinson’s disease. According to the most commonly used Bazett’s formula, 12.9% of patients had potentially dangerous QT prolongation; however, the number was only 4.0% when the Framingham formula was used. There was only moderate agreement between both formulas. None of the patients had QTc > 500 ms, which is associated with substantially high risk of torsade de pointes [16,18]. The Bland–Altman diagram also showed overestimation of the QT length caused by Bazett’s formula. The QTcB length was significantly affected by heart rate, which explained the greatest part of its variance. It is in agreement with previous studies and shows the imperfection of this formula [12,13]. QT interval length depends on HR. Faster HR results in shorter QT, and the correction for HR is neccesery for adequate risk stratification. Many formulas were created to correct QT interval, for example, QTcB, QTcF, Fridericia, Hodges [13,19,20]. The optimal QT correcting formula should not depend on HR; however, all commonly used formulas have some bias. The Bazett’s formula is the most commonly used; however, it undercorrects QT length if HR is below 60 per minute and overcorrects if HR is above 60 per minute [19,20]. In two studies, the reference interval in healthy adults with normal HR for QTcB was above 470 ms for males and 480 ms for females, and was much higher than usually used values (450 ms for males and 460–470 ms for females). Reference intervals for other mentioned formulas were in agreement with previously mentioned clinical standards [13,20]. In a study by Vandenberk et al., QTcF and Friderica’s formula were the best in mortality prediction in patients with HR < 90 beats per minute, so QTcF seems to be more suitable for our study [13]. QT-prolonging drugs were commonly used, but the drugs from the highest risk group were rarely prescribed in our PD group in comparison with other studies, and domperidone was not used at all [8,9]. The prevalence of long QTcB in this study seems to be lower in comparison with similar screening performed in the United Kingdom, where it was found in 21% (QTcB) of patients, which might be partially explained by differences in prescribed drugs, clinical practice and methodology [8].

Data from a review including seven prospective studies report 8.7% prevalence of QTcB ≥ 440 ms in general population [21]. In the Spanish population above 40 years of age, QTcB prolongation rate was 9.8% for cut point 440 ms [22]. On the other hand, in a study from China, 31.6% of patients over 35 years had QTcB longer than 440 ms [23]. For this cut point in our studied group, 26% and 11% of patients had long QT for QTcB and QTcF, respectively. QT prolongation was found in 6.3% of the general population in a study performed in the USA, which defined normal QT corrected with Fridericia formula as below 454 ms for men and 463 ms for women. [24]. A long QT in PD seems to be relatively common in comparison with those studies; however, two of them have similar or larger prevalence of QT prolongation. These results might be potentially explained by specific predisposition to QT prolongation associated with the disease, but older age, QT-prolonging drugs in the case of our patients, and differences in methodology probably also have a contribution.

Regression analysis in the study revealed, regardless of the used formula, that age, male gender and QT-prolonging drugs were predictive of QTc length. Heart disease, although significant for bivariate analysis, did not reach p-value below 0.05 in multiple linear regression analysis. Age has the largest effect on QTc length in our study except HR for QTcB. It is a well-known risk factor for QT prolongation [18,25,26]. Age influence was also reported in PD, but not in all studies assessing its effect [8,27]. Longer QTc interval in males was rather an unexpected finding. The literature reveals that females have longer QTc than males. However, with age this effect is diminished [25,26]. The old age of our patients and potential confounders might partially explain this effect in the study. In the study by Cunnington et al., there were more men in the group with long QTcB, but the difference was not statistically significant (*p* = 0.09) [8]. In two studies, QTc prolongation was correlated with autonomic dysfunction in the Valsalva maneuver [5,6]. The findings of some studies suggest association of male sex with earlier autonomic dysfunction and higher prevalence of orthostatic hypotension. This may have a contribution in the explanation of the male sex effect in our study; however, this hypothesis should be treated with caution [28,29]. Drugs affected QTc length in our study, which is in agreement with results reported by Cunnington et al. and Gibbons et al., but J. Piqueras-Flores et al. did not find significant drug effect on QTc prolongation in PD [4,8,11]. That results suggests that using QTc-prolonging drugs in PD need special attention because drugs had some effect in our study even if there was a relatively low number of drugs from the highest risk group. Longer QTc in PD patients in comparison with healthy controls and/or association between QTc prolongation and PD severity was previously reported [5,6,7,8,11]. On the other hand, there are also studies that did not find such association [27,30]. Interestingly, in one study there was a positive correlation between QRS and disease’s severity in HYs, which could potentially affect the QT interval [27] and seems to be consistent with the report about left ventricular hypertrophy in PD [4]. Absence of that relation between the severity and the QTc length in our study might be explained by its interference with other factors, which influenced QT length.

PD patients have an increased risk of developing cardiac pathology during the disease course. The most common is dysautonomia, but other cardiovascular dysfunctions, like cardiomyopathy, coronary heart disease, arrhythmias, and sudden cardiac death, may also occur. Cardiac autonomic dysfunction in PD may result from sympathetic or parasympathetic denervation, and postsynaptic receptor upregulation [3]. PD patients can develop coronary heart disease, especially with the presence of classical risk factors for vascular disease: atherosclerosis, diabetes, hyperlipidemia, arterial hypertension. Various types of conduction defects and arrhythmias have also been reported in PD patients, including the following: PR-interval, and QT-interval prolongation, right bundle branch block, or bradycardia. Moreover, cardiomyopathy is a rare cardiac complication in PD. Concentric remodeling and ventricular hypertrophy were reported to be associated with an increased Hoehn–Yahr staging in PD [4]. All the above-listed cardiovascular defects may lead to a sudden death due to cardiac disease, and they have also been reported in PD patients [31].

In the context of this study, an issue of many drugs prescribed for the geriatric PD patients seems to be especially important. Those PD patients, besides having problems associated with motor and nonmotor symptoms (for example depression, psychosis, dementia) significantly affecting health-related quality of life, usually also have multiple comorbidities like diabetes, frailty, sarcopenia, reduced renal function, liver dysfunction, endocrinal abnormalities, hypertension, ischemic heart disease, heart failure and other cardiovascular diseases. It causes polytherapy and may result in numerous side effects and drugs interactions, including life threatening arrhythmias caused by concomitant use of several QT-prolonging drugs [32,33,34]. Routine laboratory testing and detailed anamnesis can help in the identification of patients especially vulnerable to the dangers of QT prolongation (for example: hypothyroidism, electrolyte imbalance, liver or renal failure) and common QT-prolonging drugs interactions. ECG should always be performed before the initiation of treatment with QTc-prolonging drugs and regularly repeated during therapy [33]. The list of QT-prolonging drugs (www.crediblemeds.org, accessed on 29 March 2020) used in this study is a free tool [15]. There are also lists of drugs potentially inappropriate for treatment of the elderly population due to a risk of side effects higher than expected benefits. Those lists that can be helpful in geriatric PD population are as follows: the Beer’s Criteria, START/STOPP (Screening Tool to Alert doctors to the Right Treatment/Screening Tool of Older Persons’ potentially inappropriate Prescriptions), PRISCUS list and FORTA (Fit fOR The Aged) [33,34]. The conclusions of the last study stated that using the FORTA list might result in beneficial reduction of those drugs including QT-prolonging drugs in the group of geriatric PD patients [34].

The neurodegenerative process in PD is related with a higher risk of cardiovascular complications, and thus it is legitimate to be particularly attentive while prescribing different medication in this group of patients. Moreover, cardiovascular comorbidities in PD require more attention because they may significantly contribute to the mortality rate.

The study has some limitations. Firstly, it was impossible to have regard for all potential risk factors. For example, calcium and magnesium levels are not standard laboratory tests in the movement disorders clinic and were not assessed. Exclusion of patients with bundle branch block also could cause bias. Another reason for exclusion of some patients was the artifacts associated with a prominent tremor, which affected quality of ECG and made reliable QTc assessment impossible. We are aware that the final number of recruited PD patients was relatively low, but the study was carried out in a single center, a tertiary referral clinic, which provide comprehensive assessment for complicated cases, and every patient was examined by the same movement disorder specialist. We believe that those precautions allowed us to gathered a homogenous group, and more reliable information. There are differences in drugs prescription between medical centers and countries, and used drugs also might reflect some specific practices from our hospital, so generalizability of the findings is limited. The aim of study was the assessment of QT interval and influencing factors in a homogenous group of PD patients, so we decided not to include the control group. The lack of a control group makes it impossible to enable an accurate comparison with the Polish general population of similar age; however, a larger study with a control group and a study group extended to atypical parkinsonisms is planned.

## 5. Conclusions

In summary, our study suggests that QTc prolongation in PD patients seems to be associated with older age, QTc-prolonging drugs and probably male sex. Specific predilection for QTc prolongation in PD could not be excluded. Special vigilance and regular screening in this group of patients should be considered, particularly in older patients who take QTc-prolonging drugs. The effect of male gender is an unexpected finding, which should be treated with caution and needs explanation in further studies. QTcB is significantly affected by HR, so using the most popular Bazett’s formula should be avoided because it could lead to unnecessary drug withdrawals. Prevalence of QTc prolongation in PD might by higher than in the general population; however, further studies with a larger number of PD patients and a control group are needed to confirm that suspicion.

## Figures and Tables

**Table 1:** Patients’ characteristics.

**Patients’ Characteristics**	**Patients *n* = 101**
Males	65 (64%)
Age	64.5 ± 8.1 years
PD duration	9.3 ± 4.8 years
UPDRS ON	13 (8–19)
UPDRS OFF	37.9 ± 14.8
Hoehn-Yahr scale ≥3	62 (61%)
Hypertension	45 (45%)
Heart diseases *	16 (16%)
Diabetes mellitus	8 (8%)
Heart rate	71 ± 10.7
On QTc-prolonging drugs	56 (55%)
Long QTcB	13 (13%)
Long QTcF	4 (4%)

* Ischemic heart disease, heart failure, QTcB—QT corrected with Bazett’s formula, QTcF—QT corrected with Framingham formula, UPDRS ON and UPDRS OFF -Unified Parkinson’s disease rating scale in ON and OFF state.

**Table 2:** QTc-prolonging drugs taken by studied patients according to category.

	Drugs According to Category ^1^			
	Known	Possible	Conditional	Mean Dose
Psychiatric	7 (8%)	8 (10%)	18 (22%)	
Escitalopram	6 (7%)			21.6 mg
Quetiapine			5 (6%)	35 mg
Paroxetine			4 (5%)	20 mg
Trazodone			4 (5%)	100 mg
Sertraline			4 (5%)	50 mg
Mianserin		4 (5%)		15 mg
Venlafaxine		2 (2%)		112.5 mg
Citalopram	1 (1%)			5 mg
Mirtazapine		1 (1%)		30 mg
Clozapine		1 (1%)		25 mg
Olanzapine			1 (1%)	5 mg
Neurological	3 (4%)	1 (1%)	20 (24%)	
Donepzeil	3 (4%)			10 mg
Memantine		1 (1%)		10 mg
Amantadine			20 (24%)	235 mg
Cardiac	1 (1%)	2 (2%)	6 (7%)	
Sotalol	1 (1%)			160 mg
Hydrochlorothiazide		2 (2%)		18.8 mg
Indapamide			6 (7%)	1.7 mg
Urological	0	2 (2%)	2 (2%)	
Tolterodine		2 (2%)		2 mg
Solifenacin			2 (2%)	5 mg
Gastrointestinal	0	0	13 (16%)	
Pantoprazole			10	22 mg
Omeprazole			2 (2%)	20 mg
Lansoprazole			1 (1%)	30 mg
Sum = 83 (100%)	11 (13%)	13 (16%)	59 (71%)	

^1^ QTc-prolonging drugs categorized according to QT-prolonging drugs list.

**Table 3:** Characteristics of patients with long QTcF and/or QTcB.

Sex, Male = 1	Age (Years)	Drugs (mg/d)	Diabetes	Hypertension	Heart Disease	HYs	UPDRS ON	UPDRS OFF	PD Duration (Years)	QTcB (ms)	QTcF (ms)
1	70	Sotalol 160 Indapamide 1.5	1	1	0	3	19	49	10	450	460
1	73	Quetiapine 25	0	0	0	2	5	27	7	482	458
1	70	Sertraline 50	0	0	1	3	5	24	11	491	484
1	68	Pantoprazole 20 Trazodone 100	0	1	1	4	24	56	13	475	461
1	59	Pantoprazole 20 Paroxetine 20	0	1	0	3	16	43	18	479	444
1	69	Sertraline 50	0	1	1	5	37	58	8	478	432
1	66	Tolteradine 2 Amantadine 200	0	1	0	3	12	47	11	453	432
1	64	Paroxetine 20 Trazodone 150	0	1	0	3	19	34	17	462	447
1	60	Tolteradine 2	0	0	0	5	31	68	6	452	436
1	55	-	0	1	0	3	15	54	10	452	430
1	79	Memantine 10 Pantoprazole 20	0	0	1	3	16	30	10	456	423
1	66	-	0	0	0	2	4	12	2	460	429
0	71	-	0	1	0	4	25	57	15	469	441

The upper part of the table shows patients with long QTcF and QTcB, the lower with long QTcB and normal QTcF. Abbreviations: HYs—Hoehn Yahr scale, UPDRS—Unified Parkinson’s Disease Rating Scale, PD—Parkinson’s disease.

**Table 4:** Bivariate analysis for QTcB and QTcF.

	QTcB		QTcF	
Assessed Factor	Comparisons and Correlations	*p*	Comparisons and Correlations	*p*
Male vs. female	427.0 ± 24.5 vs. 422.7 ± 17.9	0.312	417.7 ± 20.6 vs. 409.5 ± 15.1	0.026 *
With vs. without QTc-prolonging drugs	429.2 ± 24.3 vs. 420.9 ± 19.0	0.062	417.7 ± 21.3 vs. 411,1 ± 15.7	0.073
With vs. without hypertension	427.7 ± 23.9 vs. 423.8 ± 21.1	0.384	418.4 ± 19.3 vs. 411.9 ±18.8	0.092
With vs. without heart diseases	439.1 ± 24.2 vs. 423.0 ± 21.2	0.008 *	426.3 ± 21.8 vs. 412.6 ± 18.0	0.008 *
With vs. without diabetes	432 (414–434) vs. 423 (408–440)	0.791	415 (400–425) vs. 407 (398–428)	0.673
HYs ≥ 3 vs. HYs < 3	428.3 ± 23.0 vs. 421.0 ± 20.8	0.109	416.0 ± 19.6 vs. 412.7 ± 18.6	0.396
UPDRS ON	R_S_ = 0.13	0.185	R_S_ = 0.10	0.312
UPDRS OFF	R_P_ = 0.05	0.615	R_P_ < 0.01	0.965
Age	R_P_ = 0.29	0.003 *	R_P_ = 032	0.001 *
PD duration	R_P_ = 0.07	0.512	R_P_ = 0.06	0.557
HR	R_P_ = 0.42	<0.001 *	R_P_ = -0.06	0.576

*—statistically significant results, HYs—Hoehn-Yahr scale, UPDRS—Unified Parkinson’s Disease Rating Scale, PD—Parkinson’s disease, HR—heart rate.

**Table 5:** Final regression models for QTcB and QTcF.

	QTcB				QTcF			
	β	β SE	*p*	r_p_^2^	β	β SE	*p*	r_p_^2^
Male vs. female	5.85	1.99	0.004	0.083	5.45	1.83	0.004	0.083
QTc-prolonging drugs	4.32	1.86	0.022	0.053	4.00	1.75	0.025	0.051
Age	0.88	0.23	<0.001	0.132	0.84	0.21	<0.001	0.134
HR	0.97	0.18	<0.001	0.240	-	-	-	-

Β—regression coefficet, β SE—standard error of regression coefficet, r_p_^2^—coefficient of partial determination, HR—heart rate.

## Data Availability

The data presented in this study are available on request from the corresponding author. The data are not publicly available due to law restrictions [any medical data are sensitive information].
